# Impacts of extreme temperatures on mood disorders: A systematic review

**DOI:** 10.1192/j.eurpsy.2025.10110

**Published:** 2025-09-29

**Authors:** Navya Manoj, Mary Katharine Kennedy, Michelle Liu, Andrew Toyin Olagunju

**Affiliations:** 1 Michael G. DeGroote School of Medicine, McMaster University, Hamilton, ON, Canada; 2 Department of Psychiatry and Behavioural Neurosciences, McMaster University, Hamilton, ON, Canada; 3 Forensic Psychiatry Program, St. Joseph’s Healthcare Hamilton, Hamilton, ON, Canada; 4 Department of Psychiatry and Behavioral Sciences, University of Oklahoma, Oklahoma City, OK, USA; 5 Discipline of Psychiatry, The University of Adelaide, Adelaide, SA, Australia

**Keywords:** bipolar disorder, climate change, depression, extreme temperature, mental health

## Abstract

**Background:**

Climate change has contributed to an increase in extreme temperatures globally, with mounting evidence suggesting a relationship between extreme temperature exposure and mental health. This review synthesizes findings on the impacts of extreme temperatures on several aspects of mood disorders.

**Methods:**

This systematic review was conducted following the Preferred Reporting Items for Systematic reviews and Meta-Analyses guideline. Major databases (MEDLINE/PubMed, PsycINFO, Scopus, and Web of Science) were searched for eligible studies. Study quality was assessed using the Joanna Briggs Institute critical appraisal tool.

**Results:**

From the 471 identified reports, 22 were included in the final review. The included studies were set in Asia (*n* = 8), North America (*n* = 7), Europe (*n* = 5), and Oceania (*n* = 2), encompassing diverse designs (case-crossover, cohort, and cross-sectional). High temperatures were linked to increased hospital admissions for mood disorders, especially among adolescents, women, and the elderly. Seventeen studies identified significant correlations between extreme heat and emergency department visits, whereas others reported minimal associations. Short-term exposure to humidity was a risk factor for increased mood disorder symptoms. Extreme cold exposure was associated with increased outpatient visits and heightened symptom severity for depressive disorders, particularly among older adults and females. The included studies were generally of moderate quality.

**Conclusions:**

The evidence from this review underscores the need for multi-pronged interventions, innovative practices, and public health strategies – including urban planning, patients’ and public education, use of telemedicine, and policy measures – to mitigate the mental health consequences of climate change-driven extreme temperature events.

## Background

The prevalence of extreme temperatures is rising globally, primarily due to climate change [1, 2]. The periods of extreme temperatures are characterized by unusually high or low ambient temperatures relative to local norms, and include heat waves (with temperatures above the 90th or 95th percentile) and cold spells (with temperatures below the 5th or 10th percentile), albeit these temperature thresholds vary by region and context [1]. Climate models project that the frequency, intensity, and duration of extreme temperature events will continue to increase, posing growing risks to public health [2, 3]. Currently, around 30% of the global population experiences harmful climatic conditions – including periods of elevated temperatures and humidity – for at least 20 days per year [2]. Closely linked is the fact that heatwaves were linked with about 8%–15% increase in emergency department (ED) visits above the baseline, whereas cold waves were associated with a 5%–12% rise in emergence unit visits [3].

Several lines of evidence have highlighted the negative impacts of climate change on mental health, and there is an increasing emphasis on climate change as a multifactorial issue with both direct and indirect effects on mental health through a complex but interconnected pathophysiological mechanism [3]. Notably, pollution – particularly air pollution – plays a significant role in shaping climate change, and its impacts on human health and well-being through detrimental effects on environmental conditions and global warming [3]. While often considered distinct, pollution and climate change may represent two sides of the same coin, as pollutants such as fine particulate matter and greenhouse gases have not only direct impacts on multiple aspects of human health (e.g., respiratory, cardiovascular, and brain) but also indirect impacts through their influence on climate, especially atmospheric temperatures and the occurrence of extreme weather events [3–5]. In particular, exposure to pollution has been linked to adverse mental health outcomes, including increased risk of depression, anxiety, and cognitive decline, further highlighting its relevance to the focus of this paper. Emerging evidence has implicated an increased exposure to environmental pollutants (such as fine particulate matters [PM2.5], nitrogen oxide [NO₂], and ozone [O₃]) as one of the plausible explanations through which climate change and pollution have independent or joint impacts on brain health [4, 5]. A recent umbrella review reported that both climate hazards and air pollution were associated with a range of mental health outcomes, including mood disorders, via identifiable pathophysiological mechanisms that involve neuroinflammation, oxidative stress, and neuroendocrine disruption [4]. Another review of epidemiological and neuroimaging data also linked macroenvironmental exposures to structural and functional brain changes affecting emotional regulation and psychiatric vulnerability [5]. These shared biological pathways – including inflammation, oxidative stress, hypothalamic–pituitary–adrenal (HPA) axis dysregulation, neurotransmitter imbalance, and hippocampal structural changes – may offer mechanistic insight into how environmental pollution and extreme temperatures converge to exacerbate psychiatric symptoms and mental well-being [4, 5]. Including this broader context may enrich discussions and provide unique insight into climate-linked mental health risks [3–5].

Within this context, extreme temperatures are increasingly recognized as both physiological and psychosocial risk factors that may trigger or worsen symptoms and other outcomes in individuals with mood disorders [6, 7]. In support of this risk formulation, heat exposure was associated with cognitive impairment, irritability, and symptom decompensation, whereas cold exposure has been linked to depressive symptoms and heightened stress responses in individuals with mental illness, including those with mood disorders [6, 7]. Beyond these direct effects, extreme temperatures can also indirectly affect mental health and well-being by disrupting sleep, impairing daily functioning, reducing opportunities for social interaction, and increasing economic stress through higher energy demands or lost productivity [6–8].

Mood disorders – including depression and bipolar disorder – are associated with significant morbidity, functional impairments, and disability [7–11]. In 2019, mood disorders were one of the leading contributors to the 125.3 million disability-adjusted life years attributed to mental disorders globally [7]. Similarly, the estimates of the prevalence of mood disorders (ranging from 3.3% to 21.4%, depending on the country) are modestly high globally [12]. Considering the recent trends in extreme temperature events, experts have warned that the burden of mood disorders is likely to grow as climate change accelerates [8–11]. However, existing studies vary widely in methodological quality, geographical focus, reported outcomes, and population characteristics [8–11, 13–31], thereby leading to major gaps in the understanding of the relationship between extreme temperatures and mood disorders. Furthermore, the effects of the interaction between environmental exposures to extreme weather and social determinants of health – such as income, housing, and healthcare access – among individuals with mood disorders remain underexplored.

Given the rising frequency and intensity of extreme temperature events, this review systematically synthesizes existing evidence on their impact on mood disorders, focusing on key outcomes such as symptom severity, hospitalizations, ED visits, outpatient utilization, and adverse events. Ultimately, this study aims to elucidate evidence to inform public health strategies, clinical practice, and future research to mitigate the mental health consequences of climate change on mood disorders, particularly for vulnerable populations.

## Methods

This systematic review adhered to the Preferred Reporting Items for Systematic Reviews and Meta-Analyses (PRISMA) guideline [32] to synthesize findings on the impacts of extreme temperatures on mood disorders across diverse populations and settings. Initial searches were conducted on August 18, 2024, and an updated search and analysis occurred in March 2025. The study protocol was registered on the Open Science Framework (https://doi.org/10.17605/OSF.IO/24EMY). All databases were searched from inception to March 2025 to ensure comprehensive coverage of the literature and inclusion of recent studies.

### Definition of extreme temperatures

In this review, extreme temperatures were broadly defined to include exposure to atypical environmental temperature conditions, such as heatwaves, extreme cold, or unusual humidity levels [3]. Recognizing that temperature thresholds vary geographically and temporally, we accepted study-specific definitions of “extreme” that reflected regional and historical baselines, as reported by the original investigators.

### Search strategy

The research question was broken down into key concepts to perform a systematic database search and identify a comprehensive list of eligible reports that met the inclusion criteria [33]. MEDLINE/PubMed, PsycINFO, Web of Science, and Scopus were searched. The search strategy (additional details are included in Supplementary Table 1) was developed using strings of search words based on primary concepts [33, 34]. Each database was searched with appropriate controlled vocabulary or Medical Subject Headings, and an explosion was applied only when all subterms were relevant to the research question [33]. Free-text search terms and keywords aligned with the main search concepts [33, 34]. The final search strategy for each was documented following the PRISMA guideline [32]. Database search results were supplemented by a “snowball” search of relevant articles. Search results were uploaded to Covidence [35] for screening.

### Eligibility criteria

This review included studies focused on individuals with mood disorders exposed to extreme temperatures – such as heatwaves, cold, or humidity – using study-specific definitions reflecting regional and historical contexts. Eligible studies reported on outcomes, such as hospitalizations, ED visits, outpatient visits, symptom severity, or adverse events, with full texts available. Studies were excluded if they focused on individuals without mood disorders, non-mood psychiatric conditions, irrelevant exposures or outcomes, or were non-original studies (e.g., reviews, protocols, or editorials).

### Screening

Following the removal of duplicate reports, screening was conducted using Covidence [35], which allowed screeners to decide whether an article was “included” or “excluded.” Articles were screened based on their titles and abstracts, and the reviewers were blinded to the decision of others. At least two independent investigators (N.M., M.K.K., and M.L.) performed title and abstract screening followed by a full-text review. Articles with conflicting decisions were automatically marked for resolution. Conflicts were resolved through discussion among investigators and consultation with the senior author (A.T.O.) when necessary.

### Data extraction

Data extraction was conducted in parallel by three team members (N.M., M.K.K., and M.L.), with each article reviewed independently by two extractors and overseen by the senior author (A.T.O.) through regular consultation. Extracted data were compiled in Google Sheets by N.M. Key details collected included publication information, study design, region, population size, exposure definitions, variables analyzed, mood disorder types, outcomes measured, methods of collecting data on extreme temperature, and statistical methods. Primary outcomes – hospital admissions, ED visits, outpatient visits, symptom severity, and adverse events – were defined according to each study’s criteria. The information collected on the methods in individual reports that were included in the final review is presented in Supplementary Table 2.

### Quality assessment

To do a quality assessment of the studies included in this review, the Joanna Briggs Institute (JBI) critical appraisal tool was utilized. The JBI checklist includes specific criteria tailored to different study designs, which helps in systematically evaluating the methodological quality of studies [35]. The assessment was completed independently by N.M., M.K.K., and M.L. during data extraction. In cases where discrepancies arose between reviewers, disagreements were first discussed between the two extractors. If consensus could not be reached, the issue was reviewed and resolved through discussion with the senior author (A.T.O.) to ensure consistency and methodological rigor.

### Data analysis and synthesis

At least two independent authors (N.M., M.K.K., and M.L.) conducted the synthesis, with guidance and consultation from a senior author (A.T.O.). Findings in the included studies were grouped into five main categories: hospital admissions, ED visits, outpatient visits, symptom severity, and adverse events. Key themes such as mood disorder type, temperature extremes, regional differences, and potential mediating factors were identified.

## Results

### Selection of eligible studies

**Figure 1.**
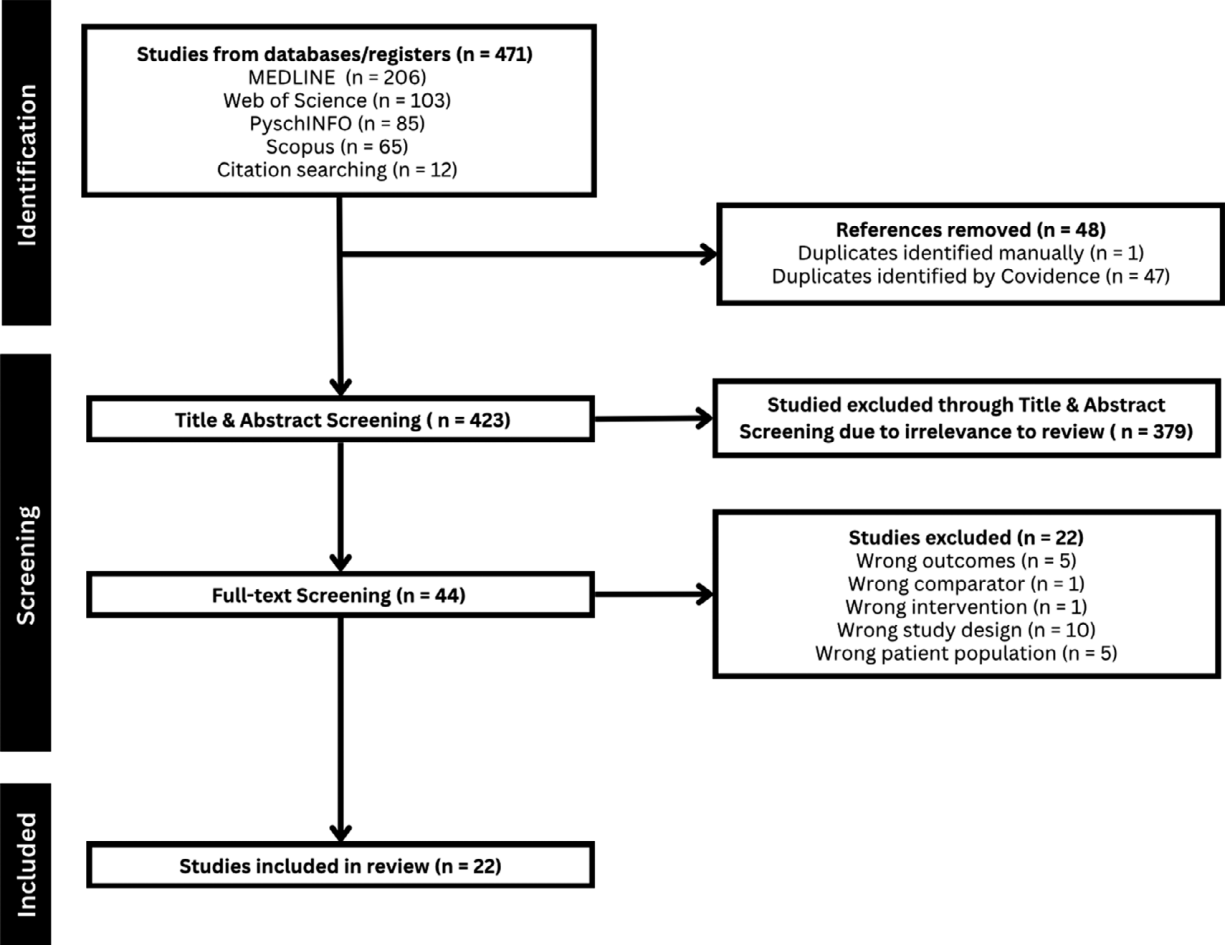
PRISMA flow diagram.

The initial database search produced 459 reports, while snowball searching yielded a total of 12 articles, yielding a total of 471 articles. Of these articles, 48 were duplicates, leading to a total of 423 articles being screened by title and abstract. After title and abstract screening, 44 articles were eligible for full-text review. From the full-text review, 22 articles did not meet the eligibility criteria, leading to 22 studies being included in the final review [9–11, 13–31]. See Figure 1 for the article selection process using a PRISMA flow diagram.

### Characteristics of the included studies

Table 1 presents findings from the included studies. All studies included in this review were observational. This included 10 time-series studies [10, 11, 13, 16, 19, 20, 27–29, 31], 7 case-crossover studies [14, 18, 21–23, 25, 30], 3 cohort studies [17, 24, 26], 1 case-control study [9], and 1 cross-sectional study [15]. Of the included studies, eight were conducted in Asia [10, 11, 17, 19, 24, 26, 27, 30, 31], seven in North America [14, 18, 21–23, 28, 29], five in Europe [9, 13, 20, 25], and two in Oceania [15, 16]. Looking at when these studies were published, 12 were published between 2020 and 2024 [10, 13, 14, 17, 18, 21–23, 25, 29–31], 8 between 2010 and 2019 [9, 11, 15, 19, 20, 26–28], and 2 between 2000 and 2009 [16, 24]. Finally, when analyzing the mood disorder outcomes reported in these studies, nine studies focused on ED visits [9, 13, 14, 18, 19, 22, 23, 28, 29], eight on hospital admissions [10, 11, 16, 20, 21, 24, 26, 27], two on outpatient visits [30, 31], two on symptom severity [15, 17], and one on adverse events [25]. As is common in environmental epidemiology, these designs allow for the investigation of population-level associations but are limited in their ability to infer causality. Supplementary Table 2 provides further details on the methods of each study.

**Table 1.** Findings from the studies included in this review

Author, year of publication	Study design	Country, region	Sample size and characteristics, if any	Extreme temperature type	Mood disorder observed	Outcomes measured	Measure of association	Results^a^ and confidence interval	Main findings
Aguglia et al., 2019 [9]	Case control	Italy, Europe	730	Extreme heat	Bipolar disorder	Admissions to the emergency psychiatric ward	OR	OR = 1.162; 95% CI = 1.011–1.231	Emergency psychiatric ward admissions for a primary diagnosis of bipolar disorder were significantly associated with maximum temperature and solar radiation.
Bundo et al. 2020 [10]	Time series	Switzerland, Europe	71,931	Extreme heat	All mood disorders	Daily hospitalization	RR	RR = 1.05; 95% CI = 0.99–1.11	Positive but less precise associations were also observed for mood disorders (F30–F39).
Chan et al., 2018 [11]	Time series	China, Asia	44,660	Extreme heat	Episodic mood disorders, depressive disorders	Hospital admissions	RR	Episodic mood disorder: RR = 1.34; 95% CI = 1.05–1.71	This study identified a positive correlation between temperature and mental disorder admissions in a warm subtropical region, with the strongest association observed for episodic mood disorders and a limited relationship noted for depressive disorders.
								Depressive disorder: RR = 0.95; 95% CI = 0.72–1.24	
Christodoulou et al., 2024 [13]	Time series	France, Europe	22,756	Extreme heat	All mood disorders	ED visits	RR	90th percentile: RR = 0.93; 95% CI = 0.91–0.95 95th percentile: RR = 0.92; 95% CI = 0.89–0.94 97.5th percentile: RR = 0.90; 95% CI = 0.87–0.93	Maximum temperature showed a negative correlation with mood disorders, suggesting a reduction in emergency room visits during periods of extreme heat or heat waves.
Deng et al., 2022 [14]	Case crossover	United States, North America	547,540	Extreme heat	All mood disorders	ED visits	ER	Temperature: ER = 5.4%; 95% CI = 2.4–8.5%	Temperature exhibited an immediate but short-term effect on mood disorders.
								Heat Index: ER = 2.0%; 95% CI = −0.4% to 4.4%	
Ding et al., 2016 [15]	Cross-sectional	Australia, Oceania	53,144; people aged 45 years and older	Extreme heat	Depression	High or very high distress (assessed using K10 scores 22); having been treated for depression or anxiety	ME, regression coefficient	**High distress (K10 ≥ 22)** Temperature between 25.0 and 26.3°C (91st–95th percentile): Coeff = 0.070 [−0.149 to 0.289]; ME = 0.4% [−0.8% to 1.5%] Temperature between 26.4 and 26.8°C (96th–95th percentile): Coeff = 0.373 [0.067–0.680]; ME = 2.0% [0.4%–3.6%] Temperature between 26.9 and 27.8°C (98th–99th percentile): Coeff = 0.481 [0.167–0.794]; ME = 2.6% [0.9%–4.2%] Temperature higher than 27.8°C (100th percentile): Coeff = 0.197 [−0.258 to 0.652]; ME = 1.0% [−1.4% to 3.5%] **Whether treated for depression or anxiety in last month** Temperature between 25.0 and 26.3°C (91st–95th percentile): Coeff = 0.098 [−0.126 to 0.323]; ME = 0.5% [−0.7% to 1.7%] Temperature between 26.4 and 26.8°C (96th–95th percentile): Coeff = 0.164 [−0.183 to 0.510]; ME = 0.9% [−1.0% to 2.7%] Temperature between 26.9 and 27.8°C (98th–99th percentile): Coeff = 0.112 [−0.260 to 0.484]; ME = 0.6% [−1.4% to 2.5%] Temperature higher than 27.8°C (100th percentile): Coeff = −0.268 [−0.792 to 0.257]; ME = −1.4% [−4.1% to 1.3%]	A one-unit increase in temperature was linked to a 0.2% increase in the likelihood of high or very high distress (*p* < 0.001, 99% CI: 0.1–0.3%). At extreme humidity levels (99th percentile), the marginal effect of heat on distress more than doubled to 0.5% (*p* < 0.001, 99% CI: 0.2–0.7%). Neither heat nor humidity was significantly associated with recent treatment for depression or anxiety, but humidity was found to amplify the adverse effects of heat on mental health, underscoring the need for healthcare reforms to address climate-related challenges.
Hansen et al., 2008 [16]	Time series	Australia, Oceania	No sample size provided	Extreme heat	All mood disorders	Hospital admissions	IRR	IRR = 1.091; 95% CI = 1.004–1.185	Heatwaves were associated with an increase in admissions for mood (affective) disorders.
								Age 15–64, *F* = 1.118 Age 15–64, *M* = 1.057 Age 64–74, *F* = 1.092 Age 64–74, *M* = 1.093 Age ≥ 75, *F* = 0.928 Age ≥ 75, *M* = 1.271 All ages, *F* = 1.093 All ages, *M* = 1.085	
Jin et al., 2023 [17]	Longitudinal cohort	China, Asia	5,600	Extreme heat, extreme cold	Depression; older populations	Depressive symptoms	HR	High temp: HR = 1.027; 95% CI = 1.013–1.041	Prolonged residence in regions with extreme temperatures (cold or hot) was linked to a higher risk of depressive symptoms.
								Low temp: HR = 1.023; 95% CI = 1.011–1.035	
Lavigne et al., 2023 [18]	Case crossover	Canada, North America	9,958,759	Extreme heat, extreme cold	All mood disorders	ED visits	OR	Extreme heat: OR = 0.995; 95% CI = 0.973–1.016	No statistically significant cumulative effects of heat over 0–5 days were observed for ED visits related to mood disorders.
								Extreme cold: OR = 0.985; 95% CI = 0.959–1.011	
Lee et al., 2018 [19]	Time series	South Korea, Asia	166,579	Extreme heat	Depression	Emergency admissions	RR	RR = 1.213; 95% CI = 1.013–1.491	High temperatures were associated with increased emergency admissions for depression, and clinicians are encouraged to consider the potential impact of elevated temperatures on mental health.
							AN	AN = 263.668; 95% CI =−6.819 to 534.155	
							AF	AF = 11.6; 95% CI = −0.3 to 23.5	
McWilliams et al., 2014 [20]	Time series	Ireland, Europe	No sample size provided	Extreme heat	Depression, bipolar disorder	Psychiatric admissions	*X*^2^	Mania *X*^2^: Region 1 = 3.699 Region 2 = 4.907 Region 3 = 2.587 Region 4 = 5.055 Region 5 = 10.975 Region 6 = 2.818 Region 7 = 2.314 Region 8 = 6.248 Region 9 = 2.688 Region 10 = 3.123 Region 11 = 3.713 Region 12 = 0. 317	No consistent relationship was found between temperature and admissions for affective disorders.
								Depression *X*^2^: Region 1 = 2.193 Region 2 = 1.162 Region 3 = 7.706 Region 4 = 3.597 Region 5 = 7.392 Region 6 = 0.660 Region 7 = 7.479 Region 8 = 3.809 Region 9 = 3.406 Region 10 = 6.865 Region 11 = 5.564 Region 12 = 0.389	
							*B* coefficient	Mania B coefficient: Region 1 = 0.000 Region 2 = −0.001 Region 3 = 0.002 Region 4 = 0.001 Region 5 = −0.006 Region 6 = NR Region 7 = −0.001 Region 8 = −0.001 Region 9 = 0.000 Region 10 = 0.000 Region 11 = −0.002 Region 12 = −0.001	
								Depression B coefficient: Region 1 = 0.002 Region 2 = 0.002 Region 3 = −0.001 Region 4 = NR Region 5 = −0.007 Region 6 = −0.001 Region 7 = −0.001 Region 8 = −0.003 Region 9 = 0.001 Region 10 = 0.001 Region 11 = −0.001 Region 12 = 0.001	
Niu et al., 2023 [21]	Case crossover	United States, North America	82,982; young people aged 6–25 years	Extreme heat	Depression, bipolar disorder	Mental health–related ED and hospital admissions	OR	**Depression** (6–11 y/o): OR = 1.31; 95% CI = 0.83–2.07 (12–17 y/o): OR = 1.13; 95% CI = 0.94–1.36 (18–25 y/o): OR = 1.26; 95% CI = 0.96–1.66	Adolescents diagnosed with bipolar disorder (OR: 1.39, 95% CI: 1.06–1.83) showed significant vulnerability to elevated temperatures, as did individuals with depression.
								**Bipolar** (12–17 y/o): OR = 1.39; 95% CI = 1.06–1.83 (18–25 y/o): OR = 1.37; 95% CI = 0.85–2.22	
Nori-Sama et al., 2022 [21]	Case crossover	United States, North America	3,496,762	Extreme heat	All mood disorders	ED visits	IRR	IRR = 1.07; 95% CI = 1.05–1.09	Days of extreme heat were associated with higher rates of ED visits for a composite measure of mental health diagnoses and ED visits associated with specific mental health conditions, including mood disorders.
Runkle et al., 2024 [23]	Case crossover	United States, North America	No sample size provided; pregnant individuals	Extreme heat	Depression, bipolar disorder	ED visits	IRR	**Depression** Urban (0 days): IRR = 1.03; 95% CI = 0.88–1.19 Urban (0–2 days): IRR = 1.10; 95% CI = 0.92–1.30 Urban (0–6 days): IRR = 1.13; 95% CI = 0.91–1.41 Suburban (0 days): IRR = 1.25; 95% CI = 0.82–1.89 Suburban (0–2 days): IRR = 1.21; 95% CI = 0.75–1.95 Suburban (0–6 days): IRR = 1.24; 95% CI = 0.67–2.29 Rural (0 days): IRR = 0.92; 95% CI = 0.49–1.73 Rural (0–2 days): IRR = 0.95; 95% CI = 0.46–1.95 Rural (0–6 days): IRR = 1.46; 95% CI = 0.55–3.85	For this pregnant population in the southeastern United States, short-term exposure to high ambient temperatures during the warm season was associated with a greater risk of ED visits for depression and bipolar disorder.
								**Bipolar disorder** Urban (0 days): IRR = 1.49; 95% CI = 1.06–2.06 Urban (0–2 days): IRR = 1.47; 95% CI = 0.99–2.17 Urban (0–6 days): IRR = 1.81; 95% CI = 1.08–3.03 Suburban (0 days): IRR = 1.01; 95% CI = 0.60–1.70 Suburban (0–2 days): IRR = 1.14; 95% CI = 0.60–2.16 Suburban (0–6 days): IRR = 1.05; 95% CI = 0.46–2.36 Rural (0 days): IRR = 0.39; 95% CI = 0.09–1.66 Rural (0–2 days): IRR = 0.85; 95% CI = 0.19–3.81 Rural (0–6 days): IRR = 0.85; 95% CI = 0.09–8.17	
Shapira et al., 2004 [24]	Cohort	Israel, Asia	5,153	Extreme heat	Depression, bipolar disorder	Monthly hospital admissions	Mean monthly admission rate	Bipolar disorder: *F*(3, 128) = 4.515, *p* < 0.005	Increased environmental temperatures were identified as a risk factor for major depressive episodes among bipolar disorder patients with psychiatric comorbidities in Israel.
Stivanello et al., 2020 [25]	Case crossover	Italy, Europe	48,305; aged 18 years or older	Extreme heat	Depression, bipolar disorder	Adverse events (i.e., death)	OR	Depression: OR = 1.083; 95% CI = 1.030–1.138	Individuals with depression faced the highest risk of temperature-related mortality, while associations for mania and bipolar affective disorders were not statistically significant.
								Bipolar disorder: OR = 1.027; 95% CI = 0.881–1.197	
Sung et al., 2013 [26]	Cohort	China, Asia	4,559	Extreme heat	Bipolar disorder	Hospital admissions	RR	90th–94th percentile (29.0°C): RR = 1.34; 95% CI = 1.20–1.51	Admissions for bipolar disorder were significantly associated with increasing temperatures above 24.0°C (50th percentile), particularly in adults and females.
								95th–99th percentile (29.7°C): RR = 1.29; 95% CI = 1.13–1.47	
								>99th percentile (30.7°C): RR = 1.5; 95% CI = 1.23–1.84	
Trang et al., 2016 [27]	Time series	Vietnam, Asia	21,443	Extreme heat	All mood disorders	Daily hospital admissions	RR	95th percentile of maximum temperature (>36^°^C): RR = 1.07; 95% CI = 0.82–1.40	Although mood disorders showed weak correlations to heatwaves, the risk of admissions increased during prolonged heat wave periods of three and seven consecutive days.
Wang et al., 2014 [28]	Time series	Canada, North America	No sample size provided	Extreme heat, extreme cold	All mood disorders	ED visits	RR	Heat: RR = 1.68; 95% CI = 1.17–2.40	Significant rises in hospital emergency room admissions for mood disorders were observed during high-temperature periods, with peak effects occurring at 0–7 lag days, particularly at lag 0 or lag 1. Individuals with mood disorders were more affected by high temperatures than other mental health conditions, with no notable associations found for cold temperatures.
								Cold: no data provided	
Yoo et al., 2021 [29]	Time series	United States, North America	2,893,764	Extreme heat, extreme cold	All mood disorders	ED visits	OR	Heat: RR = 1.33; 95% CI = 1.03–1.7	Short-term exposure to extreme heat (27.07°C) was associated with increased ED visits for various mental disorders, including substance abuse, mood and anxiety disorders, schizophrenia, and dementia.
								Cold: RR = 1.00; 95% CI = 0.82–1.23	
Zhang et al., 2020 [30]	Case crossover	China, Asia	1,133,222	Extreme heat, extreme cold	Depressive disorder, affective disorder	Daily hospital outpatient visits		Shenzhen (2.5th: 11°C), Lag 0–9: OR = 1.26; 95% CI = 1.18–1.35 Zhaoqing (2.5th: 11°C), Lag 0–9: OR = 1.22; 95% CI = 1.00–1.48 Huizhou (2.5th: 11°C), Lag 0–9: OR = 1.32; 95% CI = 1.08–1.62	Exposure to both extreme heat and cold heightened the risk of mental disorders, with cold temperatures having a more pronounced impact. These findings were consistent across affective disorders, anxiety, depression, schizophrenia, and organic mental disorders.
								Shenzhen (2.5th: 11°C), Lag 0–9: OR = 1.50; 95% CI = 0.94–2.36 Zhaoqing (2.5th: 11°C), Lag 0–9: OR = 1.22; 95% CI = 1.00–1.48 Huizhou (2.5th: 11°C), Lag 0–9: OR = 1.10; 95% CI = 0.89–1.38	
Zhou et al., 2023 [31]	Time series	China, Asia	155,436	Extreme heat	Depression	Depression outpatient visits	OR	OR Lag 0–14: 1.179; 95% CI = 1.081–1.286	Extremely high humidex values were linked to a higher risk of depression, particularly in females and individuals aged 60 years or older.
							AN	AN: 1709; 95% CI = 819–2577	
							AF	AF: 1.10%; 95% CI = 0.50%–1.61%	

Abbreviations: AF, attributable fraction; AN, attributable number; ED, emergency department; ER, excess risk; HR, hazard ratio; IRR, incidence rate ratio; ME, marginal effect; OR, odds ratio; RR, relative risk.

a Results of some studies are partially reported due to the extensive amount of data points reported, and some values have been independently calculated based on data available.

### Extreme temperatures and hospital admissions

Studies from Asia [11, 19, 26, 27], Oceania [16], and North America [21] reported a significant link between high temperatures and hospitalizations for mood disorders in general. Chan et al. [11] note hospital admissions specifically for episodic mood disorders (RR: 1.3; 95% CI: 1.05–1.71), but not for depressive disorders, for which no significant association was observed (RR: 0.95; 95% CI: 0.72–1.24). In this study, episodic mood disorders and depressive disorders were treated as two distinct diagnostic categories, underscoring that the observed associations applied only to the former. Niu et al. [21] reported increased hospital admissions during heat events. While the association with depression was not significant across age groups, there was a significant increase in bipolar disorder admissions among 12–17-year-olds (odds ratio [OR]: 1.39; 95% CI: 1.06–1.83), but not in the 18–25 age group (OR: 1.37; 95% CI: 0.85–2.22) [21]. Demographic factors such as age and sex appear to have some influence on these associations. Notably, Hasen et al. [16] noted that females aged 15–64 had an incidence rate ratio (IRR) of 1.118, which is notably higher compared with males aged 15–64 with an IRR of 1.057. However, when comparing the sexes aged 75 and older, males had an IRR of 1.271, and females had an IRR of 0.928 [16]. In contrast, Trang et al. [27] found no statistically significant association between extreme heat (above the 95th percentile, >36°C) and daily hospital admissions (RR = 1.07; 95% CI: 0.82–1.40).

### Extreme temperatures and ED visits

The studies included in this review highlight an association between heat waves and increased ED visits, although the strength of these associations varies, with weaker trends observed at extreme temperature percentiles [9, 10, 13, 18, 19]. Deng et al. [14] noted that across mood disorders, temperature exhibited a short-term effect on ED visits compared to the heat index. Similarly, Runkle et al. [23] note that pregnant individuals with short-term exposure to high ambient temperatures during the warm season were linked to an increased risk of ED visits for depression and bipolar disorder. The risk of depression-related visits was highest in suburban areas (IRR = 1.25 on the same day, increasing to 1.24 over 6 days) and lowest in rural regions, where the association became apparent only after prolonged exposure (IRR = 1.46 at 6 days) [23]. Similarly, bipolar disorder visits showed the strongest association in urban areas (IRR = 1.49 on the same day, rising to 1.81 over 6 days), whereas no significant risk was observed in rural settings [23].

Lavigne et al. [18] examined ED visits and reported an OR of 0.985 (95% CI: 0.959–1.011) for extreme cold exposure, suggesting no statistically significant association between extreme cold and ED visits. Wang et al. [28] also reported no notable associations between cold temperatures and ED visits, aligning with findings from the other studies.

### Extreme temperatures and outpatient visits

Looking at the impact of extreme temperatures on outpatient visits, short-term exposure to extreme temperatures, both hot and cold, has been linked to an increased risk of outpatient visits [30, 31]. Zhang et al. [30] conducted a case-crossover study examining daily hospital outpatient visits for depressive and affective disorders. The study found that exposure to both extreme cold and extreme heat increased the risk of outpatient visits, with cold temperatures having a more pronounced effect [30]. In Shenzhen, extreme cold (2.5th percentile) over a lag of 0–9 days was associated with a significant increase in outpatient visits for depressive disorders (OR = 1.26; 95% CI: 1.18–1.35) [30]. Similar associations were observed in Zhaoqing (OR = 1.22; 95% CI: 1.00–1.48) and Huizhou (OR = 1.32; 95% CI: 1.08–1.62) [30]. Zhou et al. [31] supported these findings, as extremely high humidex values were significantly associated with increased depression visits (OR = 1.179; 95% CI: 1.081–1.286), with an estimated attributable number (AN) of 1,709 visits (95% CI: 819–2,577) and an attributable fraction (AF) of 1.10% (95% CI: 0.50%–1.61%). The effect was more pronounced among females and older adults, highlighting specific vulnerable populations [31].

### Extreme temperatures and symptom severity

Extreme temperatures significantly impact the severity of symptoms in mood disorders, particularly depressive disorders. Jin et al. [17] conducted a longitudinal cohort study among 5,600 older adults and found that prolonged residence in regions with extreme temperatures – both cold and hot – was associated with an increased risk of developing depressive symptoms. Specifically, high temperatures were associated with a hazard ratio (HR) of 1.027 (95% CI: 1.013–1.041), and low temperatures with an HR of 1.023 (95% CI: 1.011–1.035) [17]. Similarly, Ding et al. [15], using K10 scores to assess psychological distress, found that a one-unit increase in temperature was linked to a 0.2% increase in the probability of high or very high distress (*p* < 0.001; 99% CI: 0.1–0.3%). This effect more than doubled to 0.5% at the 99th percentile of humidity (*p* < 0.001; 99% CI: 0.2–0.7%), suggesting that humidity may amplify the mental health effects of heat [15].

### Extreme temperatures and adverse events

When it comes to adverse events, Stivanello et al. [25] conducted a case-crossover study in Italy involving adults with pre-existing mental health conditions to examine the impact of extreme heat on adverse outcomes, including all-cause mortality. Among mental health service users, those with depression were significantly more vulnerable to heat-related all-cause mortality, with an OR of 1.083 (95% CI: 1.030–1.138) [25]. In contrast, no statistically significant association was observed for individuals with bipolar disorder (OR = 1.027; 95% CI: 0.881–1.197), including those experiencing mania [25].

### Quality assessment

The quality assessment of the studies included in this systematic review, based on the JBI critical appraisal tool [35], reveals a generally inherent moderate standard of methodological rigor, as seen in Supplementary Table 3. The majority of the studies were time series (*n* = 10) [10–13, 16, 19, 20, 27, 28, 31] or case-crossover (*n* = 7) [14, 18, 21–23, 25, 30] designs, with some cohort (*n* = 3) [17, 24, 26], case-control (*n* = 1) [9], and cross-sectional (*n* = 1) [15] studies. Sample sizes ranged widely, reflecting substantial variability in population coverage. The risk of bias was predominantly low across most studies, with only five studies rated as having moderate risk, mainly due to limited confounder adjustment or incomplete reporting of methodological details. Most studies used appropriate methods for exposure and outcome measurement, although these were not standardized across studies – temperature metrics and mental health outcomes varied, which may limit comparability. Statistical analyses were robust and appropriate in nearly all studies, enhancing the reliability of the reported effect sizes. Overall, the quality of the included studies was rated as high (*n* = 14) [10, 14, 16–19, 21, 22, 25, 27–31], good (*n* = 3) [9, 11, 23], and moderate (*n* = 5) [13, 15, 20, 24, 26], consistently presented from highest to lowest throughout the manuscript.

## Discussion

This systematic review reports current findings from extant literature, highlighting the association between extreme temperatures and several aspects of mood disorders, including increased hospital admissions, ED visits, outpatient visits, symptom severity, and adverse events [9–11, 13–31]. These results are broadly consistent with earlier reviews demonstrating temperature-related changes in mental health outcomes, particularly during heatwaves [13, 15, 21, 31]. However, this review contributes novel insights by disaggregating outcomes by setting (inpatient, ED, and outpatient) and incorporating symptom-level data, which have been underexplored in prior literature [6, 7].

A key pattern emerging from the findings is the differential effect of extreme heat and cold on mood disorder outcomes. While extreme heat showed a clear and consistent impact, cold exposure presented mixed results and was reported in fewer studies [18, 29]. This asymmetry may reflect differences in the acute versus chronic physiological stress associated with thermal extremes or the potential disparities in the sensitivity of surveillance for heat-related versus cold-related outcomes [36]. For instance, hospitalizations for mood disorders were consistently elevated during heat events across multiple regions, particularly for episodic and bipolar disorders [11, 26]. In contrast, admissions for depressive disorders showed more variability across studies, which may be due to differences in diagnostic classification, cultural help-seeking behaviors, and hospital coding systems [36, 37].

The acute mental health burden of extreme heat was reflected in increased ED utilization, particularly for depression and bipolar disorder. These spikes were more pronounced in urban and suburban areas and occurred more rapidly than in rural settings, likely due to greater heat exposure, urban infrastructure, or healthcare-seeking behaviors [14, 23, 38]. Some studies noted that risk estimates plateaued or weakened at the highest temperature percentiles, which may be indicative of behavioral adaptation, statistical ceiling effects, or exposure misclassification [9, 10, 13, 19]. Conversely, the relationship between extreme cold and ED visits remained inconsistent and largely non-significant, suggesting that cold may exert a more diffuse or delayed influence on psychiatric crises that would warrant ED visits, or that current methodologies inadequately capture these effects of cold temperatures [18, 28, 29].

Outpatient data provided further evidence of the nuanced effects of both heat and cold on mental health care-seeking behaviors. In particular, cold temperatures were more consistently associated with increased outpatient visits for depressive symptoms than with hospital or ED visits, possibly reflecting the more chronic and subacute burden of cold stress [30]. In contrast, heat – especially when measured using composite indices like humidex – also correlated with increased outpatient presentations, particularly among older adults and women [30, 31]. This finding is biologically plausible, given known age- and sex-related differences in thermoregulation and vulnerability to dehydration, sleep disturbance, and medication side effects during heat events [30, 31, 37].

Overall, age and sex emerged as important modifiers of risk to extreme temperatures. Younger adults and women aged 15–64 appeared more vulnerable to heat-related hospitalizations, potentially due to hormonal or occupational factors, whereas adults aged 75 and older faced increased vulnerability linked to comorbidities, impaired thermoregulation, and reduced adaptive capacity [3, 16, 22, 37]. This pattern was also seen in outpatient and ED visits, emphasizing the importance of intersectional factors – such as age, sex, housing, and healthcare access – in shaping vulnerability to extreme temperature events [3, 22, 37]. Moreover, socioeconomic factors such as isolation and inadequate access to cooling resources appeared to further intensify the mental health risk to extreme temperature, especially among individuals living in urban heat islands or disadvantaged settings [22, 37].

Findings on mood symptom severity showed a worsened trend during thermal extremes, highlighting the direct physiological and psychological impacts of environmental stress [3–5]. Higher temperatures and humidity levels were associated with increased psychological distress, particularly among older individuals [15, 17]. The synergistic effect of heat and humidity appears to magnify distress – possibly by impairing sleep, elevating inflammation, or disrupting circadian regulation [15, 39, 40]. While mechanistic studies remain limited, there is a growing evidence base that suggests that both acute and cumulative exposure to temperature extremes can exacerbate mood disorder symptoms through complex biological pathways, involving inflammation, oxidative stress, HPA axis dysregulation, neurotransmitter imbalance, and hippocampal structural changes [3–5, 15, 39].

In terms of adverse events, depression was significantly associated with increased mortality during extreme heat, whereas bipolar disorder showed weaker associations. This divergence may relate to differences in medication regimens, social support, or behavioral coping during thermal stress [25]. Individuals with depression may be at greater risk of isolation, poor judgment, lack of motivation to access care for both physical and mental health needs, or diminished self-care, thereby increasing susceptibility during heatwaves [25, 36]. This diagnostic heterogeneity underscores the need for targeted risk stratification and diagnosis-specific adaptations in clinical and public health planning.

Despite the overall moderate-to-high quality of the included studies, several methodological considerations must be addressed when interpreting the evidence. Many studies employed robust longitudinal designs with large samples, enhancing causal inference [10, 14, 16–19, 21, 22, 25, 27–31]. However, inconsistencies in exposure definitions (e.g., percentiles vs. absolute thresholds, and humidex vs. raw temperature), variable outcome measures (e.g., administrative data vs. self-report), and inadequate confounder adjustment introduce heterogeneity and limit cross-study comparisons [11, 13, 15, 20, 24–27]. These issues highlight the need for standardized exposure metrics and psychiatric outcome definitions to facilitate future comparative and meta-analytic studies to synthesize findings across settings. Moreover, the reliance on ecological and hospital-based data may miss subclinical symptoms or vulnerable groups outside formal care systems. Future research should also prioritize longitudinal designs and stronger confounder control to clarify causal pathways and inform climate-sensitive mental health interventions.

## Study Implications

Despite the abovementioned shortcomings, the evidence from this study indicates that extreme temperatures – particularly heat – pose a clinically relevant risk for individuals with mood disorders, albeit this risk can vary geographically and be impacted by environmental and social determinants [41–47]. The public health implications of these study findings are wide-ranging. First, there is a need to be cognizant of the significant mental health impacts of extreme temperatures and the factors that can intensify exposure and reduce adaptive capacity to extreme heat, including living in socioeconomically disadvantaged areas (such as urban heat islands), limited access to cooling, and baseline socioeconomic deprivation [18, 41, 43–47]. Furthermore, vulnerable groups – including older adults, adolescents, pregnant people, and those with severe mental illness – require special attention due to heightened physiological and behavioral risks [45–47]. Such extra attention can include early-warning systems, targeted outreach, and the provision of support tailored to age and cognitive needs [18, 41].

Scalable, interdisciplinary, and multiprong interventions are urgently needed. Such intervention requires an integrated approach that combines clinical, environmental, and public health strategies. These include psychotropic medication reviews (especially those that impair thermoregulation) during heatwaves [48, 49], hydration support, environmental modifications (e.g., central cooling/heating), and increased provider awareness of climate-sensitive exacerbations [45, 48–52]. Urban planning must prioritize cooling infrastructure and green space expansion in high-risk areas [53, 54]. Integrating mental health into climate adaptation strategies – such as Heat Action Plans and real-time psychiatric-weather surveillance systems – is essential [44, 46, 48, 55–57].

Public education and community-engaged, multisectoral approaches are critical for equitable and locally appropriate solutions. This includes culturally tailored outreach, targeted emergency response plans, and accessible materials for populations with cognitive, linguistic, or mobility barriers [44, 46, 57, 58]. Recognizing regional and demographic differences in vulnerability is key to mitigating immediate harms and promoting long-term climate resilience and justice [47].

The study findings also underscore the advantages of innovative ideas and technology in clinical practice. For instance, the use of telemedicine may play a vital role in mitigating the impacts of extreme temperatures on at-risk individuals, particularly elderly patients or those with mobility issues [22, 38, 53, 59]. By allowing for continued access to care without requiring travel during heatwaves or cold spells, virtual consultations could reduce exposure risks while maintaining continuity of mental health support [59]. Clinicians might also consider incorporating environmental risk assessments into routine care planning, particularly during seasonal transitions or in regions prone to thermal extremes [59]. This may include proactive outreach, medication adjustments, or connecting patients with community-based cooling or heating resources as part of personalized care [22, 38, 53, 59]. In addition, raising awareness among patients about the potential mental health impacts of extreme temperatures – such as heightened risk of mood disturbances, anxiety, or sleep disruption – and equipping them with coping strategies may further strengthen resilience and support overall well-being [36]. Lastly, future research is needed to guide evidence-based clinical practice and guidelines to mitigate the mental health impacts of extreme temperatures and weather events.

## Supporting information

10.1192/j.eurpsy.2025.10110.sm001 Manoj et al. supplementary material Manoj et al. supplementary material

## Data Availability

The search strategy is provided in the Supplementary Material. Full search results and data entry forms are available from the authors upon request.

## Supplementary material

The supplementary material for this article can be found at http://doi.org/10.1192/j.eurpsy.2025.10110.
