# Supplementary material for: Impacts of extreme temperatures on mood disorders: A systematic review
Source: Eur Psychiatry. 2025 Sep 29;68(1):e149. doi: 10.1192/j.eurpsy.2025.10110 (PMC12548486; doi:10.1192/j.eurpsy.2025.10110)
Supplement: Manoj et al. supplementary material [file S0924933825101107sup001.docx]

**Supplementary Materials**

### *Supplementary Table 1:* Search Strategy Used for Respective Databases

| **MEDLINE(R)** |
| --- |
| 1. Extreme Heat/ OR High Temperature/  2. ((extreme* OR temperature*) ADJ3 (cold OR frigid* OR heat* OR hot)) OR "heat wave*").tw,kf,kw.  3. 1 OR 2  4. exp Mood Disorders/  5. ((affective OR mood*) ADJ2 disorder*) OR bipolar OR "bi-polar" OR BPD OR depress* OR MDD OR mani* OR mania*).tw,kf,kw.  6. 4 OR 5  7. exp animals/ OR exp animal experimentation/ OR exp animal experiment/ OR exp models animal/ OR nonhuman/ OR exp vertebrate/ OR exp vertebrates/  8. exp humans/ OR exp human experimentation/ OR exp human experiment/  9. 7 NOT 8 [= Animal-only studies]  10. 3 AND 6 NOT 9 |
| **PyschINFO** |
| 1. (extreme heat OR high temperature OR ((extreme* OR temperature*) ADJ3 (cold OR frigid* OR heat* OR hot)) OR "heat wave").tw.  2. exp Mood Disorders/ OR ((affective OR mood*) ADJ2 disorder*) OR bipolar OR "bi-polar" OR BPD OR depress* OR MDD OR mani* OR mania*).tw.  3. exp animals/ NOT exp humans/  4. 1 AND 2 NOT 3 |
| **Web of Science** |
| TS=(“extreme heat” OR “high temperature” OR ((extreme* OR temperature*) NEAR/3 (cold OR frigid* OR heat* OR hot)) OR “heat wave”)  AND  TS=(“affective disorder*” OR “mood disorder*” OR bipolar OR “bi-polar” OR BPD OR depress* OR MDD OR mani* OR mania*)  NOT  TS=(animal* NOT human*) |
| **Scopus** |
| (TITLE-ABS-KEY("extreme heat" OR "high temperature" OR ((extreme* OR temperature*) W/3 (cold OR frigid* OR heat* OR hot)) OR "heat wave"))  AND  (TITLE-ABS-KEY("affective disorder*" OR "mood disorder*" OR bipolar OR "bi-polar" OR BPD OR depress* OR MDD OR mani* OR mania*))  AND  (NOT TITLE-ABS-KEY(animal*) OR TITLE-ABS-KEY(human*)) |

*Supplementary Table 2: Summary of Methods on Included Studies*

| **Author & Year** | **Study Design** | **Population/**  **Data Source** | **Exposure Variables** | **Outcome Variable** | **Statistical Methods** | **Confounders/**  **Adjustments** |
| --- | --- | --- | --- | --- | --- | --- |
| Aguglia et al. [9]  2019 | Case-  Control | All patients admitted to Psychiatric Inpatient Unit, San Luigi Gonzaga Hospital, Orbassano, Italy (Sep 2013–Aug 2015) | Daily meteorological data: minimum, medium, maximum temperature; maximum humidity; solar radiation; hours of sunshine | Admission for bipolar disorder (BD) vs. controls | Logistic regression analysis | Socio-demographic and clinical characteristics |
| Bundo et al. [10]  2020 | Time Series | Daily mental health hospitalizations in Bern, Switzerland (1973–2017) | Population-weighted daily mean ambient temperature (from 2.3-km gridded weather maps) | Hospitalizations for mental disorders | Conditional quasi-Poisson regression; distributed lag linear models | Stratified by age, sex, subdiagnosis, subperiod; adjusted for seasonality, long-term trends |
| Chan et al. [11]  2018 | Time Series | Daily mental disorder hospitalizations in Hong Kong (2002–2011) | Daily mean temperature, relative humidity, air pollutants | Hospitalizations for mental disorders (overall and by disease class, gender, age-group) | Poisson generalized additive models; distributed lag nonlinear models | Adjusted for seasonal trend, long-term trend, day-of-week, holiday |
| Christodoulou et al. [13]  2024 | Time Series | National emergency psychiatric admissions in France (2015–2019); weather data from national meteorological service | Heatwave events (defined by national criteria), daily temperature, humidity | Daily counts of emergency psychiatric admissions (all mental disorders) | Poisson regression, time-series analysis | Adjusted for day of week, seasonality, holidays |
| Deng et al.  [14]  2022 | Case-  Crossover | NY State ED visits for mental disorders (May–Oct 2017–2018); NYS Mesonet weather data | Solar radiation, temperature, relative humidity, heat index, rainfall duration | ED visits for mental disorders (ICD codes) | Conditional logistic regression | Adjusted for air pollution, stratified by demographic factors |
| Ding et al. 2016 [15] | Cross-Sectional | SEEF cohort (Australia, n=267,000); linked to gridded meteorological data | Daily temperature (°C), water vapor pressure (humidity, hPa) | High/very high distress (K10 ≥22), recent treatment for depression/ anxiety | Logistic regression | Adjusted for age, sex, socioeconomic status, region |
| Hansen et al. [16]  2008 | Time Series | Adelaide, Australia hospital admissions (1993–2006) | Heatwave periods (≥5 days, daily max temp ≥35°C), daily temperature | Hospital admissions for mental, behavioral, cognitive disorders | Poisson regression, threshold analysis | Adjusted for season, long-term trend |
| Jin et al. [17]    2023 | Cohort | CHARLS cohort (China, adults ≥45y, 2011–2018) | Apparent temperature, extreme temperature events (ice days, cold spells, tropical nights) | Depressive symptoms (CES-D-10 scale) | Cox proportional hazards, mixed-effects models | Adjusted for age, sex, education, income, region |
| Lavigne et al. [18]    2023 | Case-Crossover | ED visits for mental/behavioral disorders in Alberta & Ontario, Canada (2004–2020) | Daily average temperature, extreme heat/cold (percentile-based) | ED visits for mental/behavioral disorders (ICD codes) | Conditional logistic regression | Adjusted for air pollution, pre-existing mental health, deprivation, green space, urbanization |
| Lee et al. [19]  2018 | Time Series | Emergency admissions for mental diseases in 6 major South Korean cities (2003–2013) | Daily mean temperature, extreme hot temperature (99th percentile) | Emergency admissions for mental diseases (ICD-10) | Distributed lag nonlinear model (DLNM), multivariate meta-analysis | Adjusted for day of week, long-term trends, seasonality |
| McWilliams et al. [20]  2014 | Time Series | Psychiatric hospital admissions for affective disorders in Ireland (1999–2008) | Daily weather: temperature, rainfall, sunshine, humidity, barometric pressure | Daily admissions for affective disorders | Poisson regression, time-series analysis | Adjusted for day of week, month, year, holidays |
| Niu et al.  [21]  2023 | Case-Crossover | ED/hospital encounters for mental health (ages 5–24) in New York City (2005–2011) | Daily maximum temperature, heatwave events | ED/hospital encounters for mental health (ICD-9/10) | Poisson regression, distributed lag nonlinear models | Adjusted for air pollution, day of week, holidays, seasonality |
| Nori-Sama et al. [22]  2022 | Case-Crossover | US adults, ED visits for mental health (2010–2019, May–Sept) | Daily maximum temperature (PRISM model), extreme heat (95th percentile) | ED visits for any/specific mental health conditions | Distributed lag nonlinear models, conditional logistic regression | Adjusted for relative humidity, federal holidays, time trends |
| Runkle et al. [23]  2024 | Case-Crossover | Pregnant women, psychiatric ED visits in North Carolina (2016–2020) | Daily mean temperature, warm ambient temperature | Psychiatric ED visits during pregnancy | Conditional logistic regression | Adjusted for air pollution, day of week, holidays, seasonality |
| Shapira et al. [24]  2004 | Cohort | Bipolar depressed patient admissions, Israel (1997–2001) | Monthly maximal environmental temperature, season | Monthly admissions for bipolar depression | Correlation analysis, regression | Not specified |
| Stivanello et al. [25]  2020 | Case-Crossover | Summer deaths with mental health disorders, Emilia-Romagna, Italy (2004–2017) | Daily mean temperature, heatwave events | Summer mortality in mental health disorder patients | Conditional logistic regression | Adjusted for air pollution, holidays, seasonality |
| Sung et al.  [26]  2013 | Cohort | Psychiatric inpatients with bipolar disorder, Taiwan (2000–2009) | Daily mean temperature, season | Bipolar disorder admissions | Poisson regression, time-series analysis | Adjusted for age, sex, urbanization, holidays |
| Trang et al.  [27]  2016 | Time Series | Hospital admissions for mental disorders, Hanoi, Vietnam (2008–2012) | Heatwave events (≥3 days, temp ≥95th percentile) | Hospital admissions for mental disorders (ICD-10) | Poisson regression, distributed lag models | Adjusted for day of week, holidays, seasonality |
| Wang et al.  [28]  2014 | Time Series | ER visits for mental and behavioral disorders in Toronto, Canada (2002–2010) | Daily mean temperature (hot/cold extremes, percentiles) | ER visits for mental and behavioral disorders (ICD-10) | Poisson regression with distributed lag nonlinear model (DLNM) | Seasonality, humidity, day of week, air pollutants |
| Yoo et al.  [29]  2021 | Time Series | 2.8 million ER visits for mental disorders, New York State (2009–2016) | Daily average temperature, extreme heat (region-specific percentiles) | ER visits for mental disorders (ICD-9/10) | Quasi-Poisson GLM with DLNM; meta-analysis for pooled estimates | Day of week, precipitation, long-term and seasonal trends |
| Zhang et al.  [30]  2020 | Case-Crossover | Psychiatric hospital records, 3 subtropical Chinese cities (all ages) | Daily mean temperature (city-specific percentiles) | Hospital admissions for 5 mental disorder categories | Case-crossover with DLNM, stratified by age/gender | Not explicitly stated, but design controls for time-invariant confounders |
| Zhou et al.  [31]  2023 | Time Series | 155,436 outpatient visits for depression, Chongqing, China (2014–2019) | Humidex (combined temperature and humidity), extreme high humidex (≥40) | Outpatient visits for depression | DLNM; hierarchical analysis by age and gender | Seasonality, long-term trend, day of week, holidays |
| *Abbreviations*  *BD – Bipolar Disorder*  *CES-D-10 – Center for Epidemiologic Studies Depression Scale (10-item short form)*  *CHARLS – China Health and Retirement Longitudinal Study*  *°C – Degrees Celsius*  *DLNM – Distributed Lag Nonlinear Model*  *ED – Emergency Department*  *GLM – Generalized Linear Model*  *hPa – Hectopascal (unit of pressure, used for humidity/water vapor pressure)*  *ICD – International Classification of Diseases*  *ICD-9 / ICD-10 – 9th / 10th Revision of the International Classification of Diseases*  *K10 – Kessler Psychological Distress Scale (10-item)*  *NYS Mesonet – New York State Mesonet (weather monitoring network)*  *PRISM – Parameter-elevation Regressions on Independent Slopes Model (US high-resolution climate model)*  *SEEF – 45 and Up Study Social, Economic, and Environmental Factors Study (Australia, large cohort)* | | | | | | |

*Supplementary Table 3: Quality assessment using JBI critical appraisal tool*

| **Author & Year** | **Study Design** | **Sample Size** | **Bias Risk** | **Appropriate Methods** | **Confounders Considered** | **Measurement of Outcome** | **Statistical Analysis** | **Overall Quality** |
| --- | --- | --- | --- | --- | --- | --- | --- | --- |
| Aguglia et al. [9]  2019 | Case-  Control | 730 | Low | Yes | Partially | Yes | Yes | Good |
| Bundo et al. [10]  2020 | Time Series | 71,931 | Low | Yes | Yes | Yes | Yes | High |
| Chan et al. [11]  2018 | Time Series | 44,660 | Low | Yes | Partially | Yes | Yes | Good |
| Christodoulou et al. [13]  2024 | Time Series | 22,756 | Moderate | Yes | No | Yes | Yes | Moderate |
| Deng et al.  [14]  2022 | Case-  Crossover | 547,540 | Low | Yes | Yes | Yes | Yes | High |
| Ding et al. 2016 [15] | Cross-Sectional | 53,144 | Moderate | Yes | No | Yes | Yes | Moderate |
| Hansen et al. [16]  2008 | Time Series | N/A | Low | Yes | Yes | Yes | Yes | High |
| Jin et al. [17]    2023 | Cohort | 5,600 | Low | Yes | Yes | Yes | Yes | High |
| Lavigne et al. [18]    2023 | Case-Crossover | 9,958,759 | Low | Yes | Yes | Yes | Yes | High |
| Lee et al. [19]  2018 | Time Series | 166,579 | Low | Yes | Yes | Yes | Yes | High |
| McWilliams et al. [20]  2014 | Time Series | N/A | Moderate | Yes | No | Yes | Yes | Moderate |
| Niu et al.  [21]  2023 | Case-Crossover | 82,982 | Low | Yes | Yes | Yes | Yes | High |
| Nori-Sama et al. [22]  2022 | Case-Crossover | 3,496,762 | Low | Yes | Yes | Yes | Yes | High |
| Runkle et al. [23]  2024 | Case-Crossover | N/A | Moderate | Yes | Yes | Yes | Yes | Good |
| Shapira et al. [24]  2004 | Cohort | 5,153 | Moderate | Yes | No | Yes | Yes | Moderate |
| Stivanello et al. [25]  2020 | Case-Crossover | 48,305 | Low | Yes | Yes | Yes | Yes | High |
| Sung et al.  [26]  2013 | Cohort | 4,559 | Moderate | Yes | No | Yes | Yes | Moderate |
| Trang et al.  [27]  2016 | Time Series | 21,443 | Low | Yes | Yes | Yes | Yes | High |
| Wang et al.  [28]  2014 | Time Series | N/A | Low | Yes | Yes | Yes | Yes | High |
| Yoo et al.  [29]  2021 | Time Series | 2,893,764 | Low | Yes | Yes | Yes | Yes | High |
| Zhang et al.  [30]  2020 | Case-Crossover | 1,133,222 | Low | Yes | Yes | Yes | Yes | High |
| Zhou et al.  [31]  2023 | Time Series | 155,436 | Low | Yes | Yes | Yes | Yes | High |
